# The Role of Astaxanthin as an Antioxidant and Anti-Inflammatory Agent in Human Health: A Systematic Review

**DOI:** 10.3390/ijms27020700

**Published:** 2026-01-09

**Authors:** Giuseppina Malcangi, Angelo Michele Inchingolo, Lucia Casamassima, Irma Trilli, Laura Ferrante, Marialuisa Longo, Francesco Inchingolo, Grazia Marinelli, Andrea Palermo, Gianna Dipalma, Alessio Danilo Inchingolo

**Affiliations:** 1 Interdisciplinary Department of Medicine, University of Bari “Aldo Moro”, 70124 Bari, Italy; giuseppinamalcangi@libero.it (G.M.); lucia.casamassima@uniba.it (L.C.); trilliirma@gmail.com (I.T.); lauraferrante79@virgilio.it (L.F.); dott.marialuisa.longo@gmail.com (M.L.); francesco.inchingolo@uniba.it (F.I.); graziamarinelli@live.it (G.M.); alessiodanilo.inchingolo@uniba.it (A.D.I.); 2 Department of Biomedical, Surgical and Dental Sciences, Milan University, 20122 Milan, Italy; 3 Department of Experimental Medicine, University of Salento, 73100 Lecce, Italy; andrea.palermo@unisalento.it

**Keywords:** astaxanthin, antioxidant, anti-inflammatory, oxidative stress

## Abstract

This systematic review aimed to summarize the effects of astaxanthin (ASX) supplementation on oxidative stress, inflammation, and metabolic regulation in human studies. A systematic search was conducted in Scopus, Web of Science (WOS), and PubMed for articles published between 2020 and 2025. Fifteen studies involving human participants were included, while in vitro and animal studies were excluded. ASX consistently reduced pro-inflammatory cytokines (IL-6, TNF-α, TGF-β1) and oxidative stress indices while increasing antioxidant capacity (SOD, TAC). Combined ASX and exercise interventions improved body composition, lipid profiles, insulin sensitivity, and immune recovery. In women with Polycystic Ovary Syndrome (PCOS) or endometriosis, ASX downregulated endoplasmic reticulum stress–related apoptotic pathways and improved oocyte and embryo quality. Cardiometabolic and respiratory outcomes showed improved endothelial function and reduced disease severity. Astaxanthin demonstrates broad antioxidant and anti-inflammatory properties, supporting its role as a promising adjunctive therapy for metabolic, reproductive, and cardiovascular health. Further well-designed clinical trials are needed to confirm optimal dosing and mechanisms of action.

## 1. Introduction

### 1.1. Carotenoids and Natural Therapies

**Figure 1:**
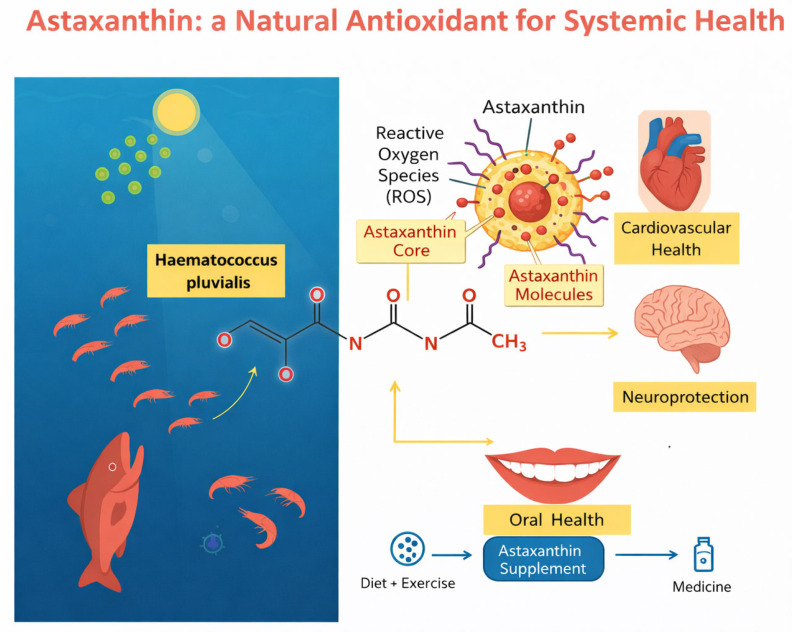
Schematic representation of the natural origin of astaxanthin and its systemic health effects. On the left, the microalga *Haematococcus pluvialis* is shown as the main natural source of astaxanthin, which is transferred along the aquatic food chain. On the right, an illustrative model of a functionalized nanoparticle is presented: astaxanthin is depicted both as molecules distributed within the nanoparticle matrix and as an astaxanthin-rich core, responsible for scavenging reactive oxygen species (ROS). The antioxidant activity of astaxanthin is associated with benefits for cardiovascular health, neuroprotection, and oral health, highlighting its potential nutraceutical and therapeutic applications.

Carotenoids represent a family of naturally occurring pigments synthesized by plants, algae, bacteria and fungi [1,2,3,4,5]. These compounds are gaining increasing attention in the field of preventive and integrative medicine because they are accessible via diet or supplementation and often exhibit favorable safety profiles. Among these, astaxanthin is distinguished by a unique chemical structure, a keto-carotenoid (xanthophyll) bearing hydroxyl and keto functional groups at each end of its polyene chain, which confers exceptional antioxidative efficacy [6,7,8]. Its natural occurrence in microalgae (such as *Haematococcus pluvialis*), krill, shrimp and salmon underscores its marine origin and highlights the concept of leveraging “marine-derived natural products” in human health [9,10]. In the broader context of natural medicine, there is a growing trend toward identifying bioactive compounds that are as close as possible to their dietary or naturally derived forms rather than entirely synthetic molecules [11,12,13,14]. This reflects a philosophy of supporting physiological homeostasis via compounds that complement endogenous defense systems, rather than overriding them with high-dose pharmaceuticals [15,16,17,18,19]. Astaxanthin (ASX), by virtue of its structural similarity to other xanthophylls and its presence in the food chain, fits this paradigm of a “natural intervention” or nutraceutical [20,21,22] that might bridge the gap between lifestyle intervention (diet, exercise) and pharmacologic treatment (Figure 1).

### 1.2. Astaxanthin: Oxidative Stress, Inflammation and the Need for Complementary Natural Strategies

Oxidative stress occurs when the generation of reactive oxygen species (ROS) outpaces the body’s antioxidant defenses, leading to lipid peroxidation [23,24,25], protein and DNA damage, mitochondrial dysfunction, and the triggering of pro-inflammatory pathways [26,27,28,29]. Chronic inflammation, in turn, is intertwined with oxidative stress and contributes to the pathogenesis and progression of a wide spectrum of diseases, cardiovascular diseases, metabolic syndrome, neurodegenerative disorders, ocular ailments, and even oral/odontologic conditions such as periodontitis [30,31,32,33]. The integration of natural compounds with antioxidative and anti-inflammatory capacities therefore emerges as a logical strategy to complement standard care and lifestyle modification, particularly in preventive medicine and in fields such as dentistry, where less invasive adjunctive therapeutic options are desirable [34,35,36]. ASX’s molecular configuration allows it to traverse cellular membranes and potentially accumulate in lipid-rich tissues, thereby conferring protective effects at the cellular and sub-cellular level. It has been shown to modulate redox-sensitive transcription factors such as Nrf2 (nuclear factor erythroid 2-related factor 2) and NF-κB (nuclear factor kappa B) [37,38,39], thereby simultaneously enhancing endogenous antioxidant defenses and suppressing pro-inflammatory signaling [40,41,42]. Importantly, the concept of a “natural medicine” in this context underscores not only the origin of the compound, but also its compatibility with physiological processes and minimal side-effect profile, which is particularly relevant in general medicine and dental practice where long-term safety is paramount [43,44,45].

Hormesis is a key conceptual framework for understanding the biological activity of astaxanthin, often referred to as a “hormetic nutrient” [46,47]. Rather than acting solely as a direct antioxidant, astaxanthin exerts its protective effects through mild adaptive stress that activates endogenous defense pathways [48]. In particular, astaxanthin stimulates the Nrf2 signaling cascade, leading to the upregulation of cytoprotective and antioxidant enzymes, while concurrently inhibiting the NF-κB pathway, a central regulator of pro-inflammatory gene expression [49,50]. This dual modulation enables cells to enhance resilience against oxidative and inflammatory insults, promoting homeostatic adaptation rather than simple radical scavenging [51]. Through this hormetic mechanism, astaxanthin supports cellular protection and functional recovery in stress-related disorders, highlighting its role as a biologically active nutrient that strengthens the body’s intrinsic defense systems [52].

### 1.3. Mechanisms of Action: Antioxidant, Anti-Inflammatory and Tissue-Protective Effects

ASX’s antioxidative mechanisms are multifaceted. It directly scavenges free radicals, such as superoxide anions, hydroxyl radicals, singlet oxygen, and interrupts lipid peroxidation chains due to its conjugated double bond structure [53,54,55,56]. Moreover, ASX has been shown to activate the Nrf2 pathway, promoting the expression of endogenous antioxidant enzymes such as superoxide dismutase (SOD), catalase and glutathione-peroxidase (GPx) [57,58,59,60]. On the anti-inflammatory side, ASX inhibits the nuclear translocation of NF-κB, decreases expression of pro-inflammatory cytokines (IL-1β, IL-6, TNF-α), and interferes with MAPK signaling (p38, JNK, ERK). Interestingly, recent data indicate a direct binding interaction between ASX and IL-6, thereby disrupting the feedback loop of inflammatory amplification [61,62]. Furthermore, ASX protects mitochondrial integrity by reducing mitochondrial ROS, preserving mitochondrial membrane potential, and limiting opening of the mitochondrial permeability transition pore, thus safeguarding cellular energy metabolism and apoptotic resistance [63,64,65,66,67,68]. Collectively, these mechanisms support a paradigm in which ASX functions not simply as a passive antioxidant, but as an “active natural medicine” that modulates cellular signaling networks [69], strengthens endogenous defenses, and attenuates the pro-oxidative/inflammatory cycle [70,71,72,73].

### 1.4. Challenges, Gaps and the Natural Medicine Perspective

Despite these promising attributes, there remain important challenges in adopting ASX as a standard natural-medicine adjunct [74,75,76,77,78]. One major limitation is heterogeneity in supplementation regimens, differences in dose, formulation (esterified vs. non-esterified) [79,80], duration of intervention and study populations [81,82,83,84,85]. This heterogeneity complicates comparison across trials and limits firm conclusions regarding optimal dosing [86,87,88,89,90]. Additionally, bioavailability remains a barrier: ASX’s lipophilicity means that its absorption depends on dietary fat, matrix formulation and individual variability [91,92,93,94,95]. Moreover, long-term outcome data remains scarce. From the natural medicine standpoint, it is also critical to emphasize that supplementation should not replace foundational interventions, such as proper nutrition, exercise, oral hygiene and risk factor control [96,97,98], but rather complement them [99,100,101]. Looking ahead, future research should address dose-response relationships, long-term safety and efficacy, synergistic interactions with other nutrients or therapies. Investigations into whether ASX supplementation can reduce reliance on anti-inflammatory or antioxidant pharmaceuticals [102,103,104,105], and whether it can shift the paradigm toward more natural-based adjunctive therapies, is of particular interest [106,107,108,109,110].

### 1.5. The Role of Astaxanthin in Preventive and Adjunctive Natural Medicine

The concept of “natural medicine” emphasizes interventions derived from natural sources, with minimal processing, and with mechanisms of action that harmonize with physiological pathways [111,112,113]. ASX exemplifies this concept: derived from algae, crustaceans or fish, it integrates into cellular membranes, modulates endogenous defense systems and exerts protective effects without the toxicity profile commonly associated with synthetic drugs [114,115]. In an era where patients increasingly seek “natural” or “integrative” options [116,117,118], ASX stands out as a scientifically grounded nutraceutical that bridges the gap between diet and therapeutic intervention [119,120]. In preventive medicine, it may serve as a daily adjunct in populations at increased oxidative/inflammatory risk (e.g., ageing individuals, smokers, those with metabolic dysregulation) [121,122,123,124,125]. Through its dual antioxidant and anti-inflammatory actions, ASX may also promote tissue resilience (e.g., vascular endothelium, gingival epithelium, ocular surface) [126,127], enhance recovery (e.g., post-exercise, post-surgery) and support long-term functional preservation (e.g., cognitive, musculoskeletal and dental) [128,129,130,131]. Moreover, since it is relatively safe and well-tolerated, its inclusion as part of an integrative care model is feasible [132,133,134,135,136]. That said, the adoption of any natural medicine requires rigorous evidence, standardized formulations, patient education and appropriate integration into multidisciplinary care pathways [137,138].

### 1.6. Aim of the Study

Therefore, the aim of this systematic review is to provide a comprehensive and critical appraisal of the current human clinical evidence on ASX as an antioxidant and anti-inflammatory agent in human health. Specifically, we will examine the effects of ASX supplementation on oxidative stress biomarkers, inflammatory mediators, metabolic and cardiovascular endpoints, immune function, ocular and oral health outcomes, and safety/tolerability. Additionally, we will highlight the implications of these findings for natural medicine practice in general medical and identify gaps in knowledge that must be addressed to support its wider incorporation into integrative care.

## 2. Materials and Methods

### 2.1. Protocol and Registration

The current systematic review was conducted following the PRISMA guidelines (Preferred Reporting Items for SR and Meta-Analyses) and International Prospective Register of SR Registry procedures (PROSPERO: 1232406)

### 2.2. Search Process

The following databases were combed from January 2020 to July 2025, to search for articles published over the last 5 years: PubMed, Web of Science (WoS), and Scopus. The search strategy was developed by combining terms relevant to the study’s purpose. In the advanced search strings used in the databases (detailed search terms are given in Appendix A), the following keywords were applied using Boolean operators to combine terms pertinent to this study’s purpose (Table 1).

### 2.3. Inclusion and Exclusion Criteria

The reviewers worked in groups to assess all relevant studies that evaluated studies considering eligible if they met the following criteria:

- Open-access and written in English;
- Published within the last five years;
- Full-text articles available for review;
- Designed as randomized controlled trials (RCTs);
- Conducted on human participants;
- Included adults aged 19 years and older;
- Investigated patients with pathological or clinical conditions relevant to the research topic.

Studies that fulfilled at least one of the following criteria were excluded:

Studies were excluded if they met one or more of the following conditions:

- Preprints or unpublished manuscripts;
- Systematic reviews, meta-analyses, case reports, or case series;
- Letters to the editor, conference abstracts, or commentaries;
- Studies involving animal models;
- In vitro or laboratory-based experiments.

### 2.4. PICO Question

The PICO format is a framework used in qualitative research to structure clinical research questions. In this study, the PICO addressed the following question: “What are the mechanistic and biological effects of orally administered ASX in adults, compared with placebo or no treatment, on markers of oxidative stress, inflammation, and metabolic function? “

Population (P): Adults (≥19 years), both males and females, either healthy or diagnosed with chronic metabolic or inflammatory conditions (e.g., type 2 diabetes, polycystic ovary syndrome, obesity, cardiovascular diseases, arthritis, or ocular disorders).

Intervention (I): Oral administration of ASX, either natural or synthetic, is delivered as capsules, oils, or other lipid-based formulations.

Comparison (C): Placebo, no treatment, or standard care (e.g., dietary interventions, physical activity, or non-carotenoid supplementation).

Outcome (O): Changes in plasma or tissue biomarkers and improvements in physiological function related to oxidative stress, inflammation, and metabolism.

### 2.5. Data Processing

Five independent reviewers (L.C., I.T., L.F., M.L. and F.I.) assessed the methodological quality and risk of bias of the included studies using the Cochrane Risk of Bias 2 (RoB 2) tool. The tool evaluates five key domains such as selection, measurement validity, confounding, and data analysis. Discrepancies in scoring were resolved through discussion and consensus, with support from additional reviewers (G.M., A.D.I., A.P., G.Mn., A.M.I., and G.D.) when needed. The reviewers screened all retrieved records based on predefined inclusion and exclusion criteria. After screening, a total of 805 articles were imported into Zotero (version 6.0.36) for organization and full-text analysis.

## 3. Results

### 3.1. Selected Studies and Their Characteristics

**Figure 2:**
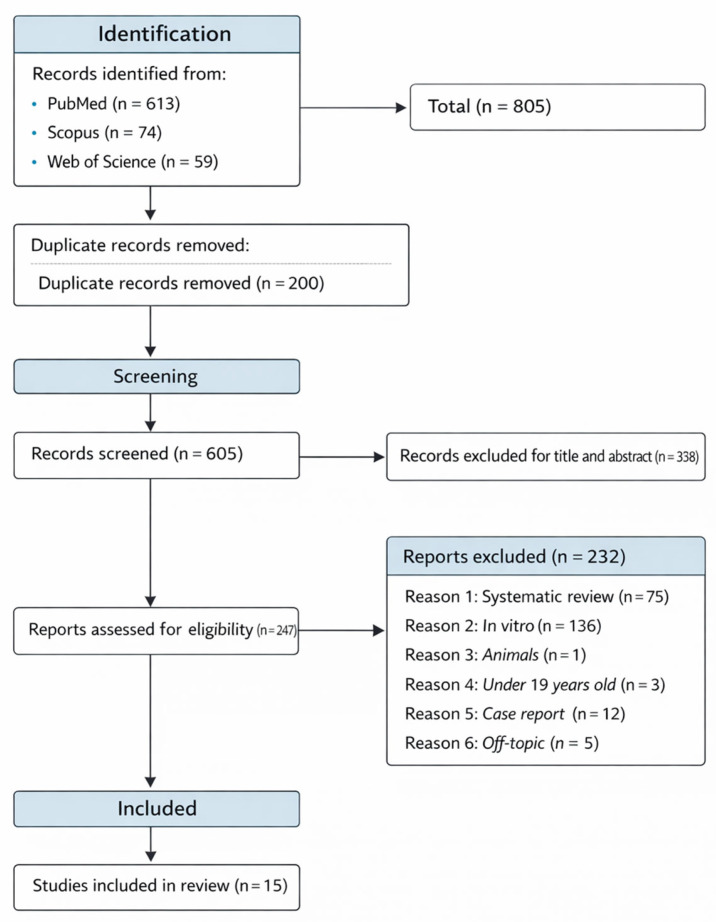
PRISMA flow diagram illustrates the study selection process, including the number of records identified, screened, assessed for eligibility, and included in the final systematic review.

The systematic search process, conducted according to PRISMA guidelines (Figure 2), identified a total of 805 records from three major databases: PubMed (*n* = 613), Scopus (*n* = 74), and Web of Science (*n* = 59). After the removal of 200 duplicates, 605 articles were screened by title and abstract. Following the application of the predefined inclusion and exclusion criteria, 247 full-text articles were assessed for eligibility. Of these, 230 were excluded for the following reasons: systematic reviews or meta-analyses (*n* = 75), in vitro studies (*n* = 136), animal studies (*n* = 1), participants under 19 years of age (*n* = 3), case reports (*n* = 12), and off-topic articles (*n* = 5). Ultimately, 15 randomized controlled trials (RCTs) met all inclusion criteria and were included in the final synthesis (Table 2).

### 3.2. Quality and Risk of Bias Assessment for the Included Articles

The methodological quality and risk of bias of the fifteen included randomized controlled trials were assessed using the Cochrane Risk of Bias 2 (RoB 2) tool (Table 3). This tool is specifically designed to evaluate potential bias in randomized clinical trials. Each study was independently reviewed by four reviewers (L.C., I.T., L.F., and M.L.), and any discrepancies in the assessments were resolved through discussion and consensus, with additional researchers (Giuseppina Malcangi., F.I., Grazia Marinelli., A.P; A.D.I., and A.M.I.) involved when necessary to ensure consistency and accuracy.

The RoB 2 tool evaluates five key domains: (1) bias arising from the randomization process, (2) bias due to deviations from intended interventions, (3) bias due to missing outcome data, (4) bias in the measurement of the outcome, and (5) bias in the selection of the reported result. Each domain was judged as “low risk,” “some concerns,” or “high risk,” leading to an overall judgment of the study’s risk of bias.

The results of the assessment showed that most of the included studies presented a low risk of bias across all domains of the Cochrane RoB 2 tool. This finding can be attributed to the adoption of rigorous experimental designs, predominantly randomized, double- or triple-blind trials, with pre-registered study protocols, clearly described randomization procedures, and minimal loss of participants during follow-up. Only one study [149] showed some concerns in the domains related to the randomization process and deviations from intended interventions, as it lacked a control group and employed a quasi-experimental design (Figure 2).

Overall, the global risk of bias (Overall) was judged to be low for the vast majority of the included trials, indicating a high methodological quality and reliability of the available evidence. A detailed summary of the item-by-item assessment for each trial is presented in Table 3, providing a comprehensive overview of the methodological rigor and potential sources of bias across the included studies. This evaluation ensures a reliable interpretation of the evidence regarding the effects of ASX supplementation in human clinical trials (Table 3).

## 4. Discussion

### 4.1. Obesity and Lipid Metabolism: A Complex Picture of Synergies and Specific Contexts

The impact of ASX supplementation on obesity and lipid metabolism is emerging as an extremely promising area of research, although the results show nuances that depend on the clinical context, the study population, and the combination with other interventions. The most robust evidence comes from studies combining ASX with physical exercise in obese subjects. Research by Saeidi et al. (2023) demonstrated that the combination of high-intensity functional training (HIFT) with 12 weeks of supplementation produced significantly greater benefits compared to the individual interventions [152]. Specifically, a marked reduction in anthropometric indices (body weight, BMI, fat percentage), an improvement in the complete lipid profile (increased HDL-C, reduced LDL-C, total cholesterol, and triglycerides), and metabolic markers (glucose, insulin, HOMA-IR) was observed. In parallel, studies by Supriya et al. (2023) and Moqaddam et al. (2024), both conducted on obese men undergoing 12 weeks of CrossFit training, further investigated the effect on adipo-myokines—the signaling molecules that orchestrate communication between adipose and muscle tissue [139,153]. Supriya et al. (2023) reported that the combination of CrossFit and ASX increased the levels of beneficial adipokines such as adiponectin and omentin-1, while reducing those associated with metabolic dysfunctions, including leptin, visfatin, vaspin, and SEMA3C [153]. This confirms the hypothesis of a synergistic action, where physical exercise stimulates the release of myokines and induces a negative energy balance, while ASX, through its antioxidant and anti-inflammatory properties (such as activating the PPARγ pathway and inhibiting NF-κB), improves insulin sensitivity and lipid metabolism [146]. Delving deeper, Moqaddam et al. (2024) documented how the combined intervention beneficially and divergently modulates the adipo-myokine profile: an increase in anabolic and protective molecules like decorin (DCN) and follistatin (FST) was recorded, along with a concurrent decrease in catabolic and pro-fibrotic factors belonging to the TGF-β superfamily, namely activin A, myostatin (MST), and TGF-β1 [139]. These findings suggest that ASX not only supports weight loss and lipid improvement but also acts at the molecular level to rebalance the hormonal environment, promoting muscle health and counteracting the chronic inflammatory processes typical of obesity.

This positive effect on the lipid profile is corroborated by studies in other populations with metabolic disorders. In the study by Ciaraldi et al. (2023) on individuals with prediabetes and dyslipidemia, ASX induced a statistically significant reduction in total cholesterol and LDL cholesterol, accompanied by an increase in HDL cholesterol [143]. This study reinforces the idea that ASX is particularly effective in subjects with an already compromised lipid profile. Similarly, the study by Jabarpour et al. (2024) on women with polycystic ovary syndrome (PCOS), a condition often associated with dyslipidemia, found a significant reduction in LDL cholesterol and an increase in HDL after eight weeks of supplementation with 12 mg/day [145]. However, the picture is not universally homogeneous. The study by Heidari et al. (2023) on patients with coronary artery disease (CAD) found no statistically significant effect on the lipid profile when comparing the ASX group to the placebo group [148]. The authors, however, provide a crucial explanation: most participants were already on statin therapy, highly potent drugs that had likely already optimized their lipid parameters. It is interesting to note that, when analyzing only the ASX-treated group, a significant reduction in total and LDL cholesterol was observed, suggesting a potential additive effect or a benefit in the absence of other therapies. This hypothesis is further supported by the results of Gonzalez et al. (2024) who found no significant differences in the lipid profiles of healthy, trained firefighters after four weeks of supplementation [141]. In this case, it is likely that the participants already had optimal lipid values at rest, leaving little room for improvement. Finally, the study by Moqaddam et al. (2024) although a research protocol for patients with heart failure, cites previous literature confirming ASX’s ability to increase HDL and decrease triglycerides, positioning it as a supplement of great interest for future cardiovascular applications [139]. In summary, ASX demonstrates robust efficacy in improving the lipid profile and metabolic parameters related to obesity, especially in populations with pre-existing metabolic dysfunctions and when combined with physical exercise. The effect appears less evident in healthy individuals or in patients already undergoing lipid-lowering drug therapies, indicating that its primary role may be as an adjuvant and preventive agent.

### 4.2. Diabetes and Glycemic Control: Molecular Mechanisms and Clinical Perspectives

**Figure 3:**
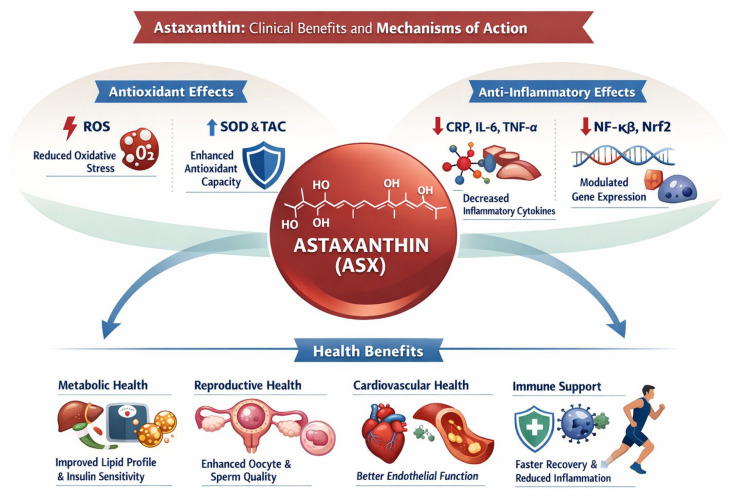
The figure provides a schematic overview of the main mechanisms and clinical effects of astaxanthin (ASX). At the center, ASX is depicted as the key bioactive compound with antioxidant and anti-inflammatory actions. On the left, its antioxidant effects are illustrated by reducing reactive oxygen species (ROS) and enhancing endogenous antioxidant defenses, such as superoxide dismutase (SOD) and total antioxidant capacity (TAC). On the right, the anti-inflammatory effects are shown by downregulating inflammatory cytokines (e.g., CRP, IL-6, TNF-α) and modulating redox- and inflammation-related signaling pathways. These molecular mechanisms are linked to beneficial clinical outcomes in metabolic, reproductive, cardiovascular, and immune health, summarized in the lower panel.

The application of ASX in the context of diabetes and prediabetes reveals significant therapeutic potential, acting through complex cellular mechanisms that go beyond simple glycemic control (Figure 3). The most emblematic study in this field is that of Sharifi-Rigi et al. (2023),which examined the effects of a 10 mg/day supplementation for 12 weeks in patients with type 2 diabetes (T2D) [140]. The results were clinically relevant, showing a significant decrease in both fasting plasma glucose (FPG) and glycated hemoglobin (HbA1c), two fundamental parameters for diabetes management. The most innovative aspect of this study lies in the analysis of the underlying molecular mechanisms. The researchers observed that ASX positively modulates the process of autophagy, a cellular “cleanup” mechanism that is often dysregulated in diabetes. Specifically, supplementation reduced the expression of the mTOR gene, a known inhibitor of autophagy, and simultaneously increased the expression of genes and proteins essential for activating the autophagic process, such as beclin-1, LC3B, Atg-5, and Atg-7. The authors hypothesize that this effect is mediated by ASX’s ability to activate AMPK protein kinase, a cellular energy sensor that inhibits mTOR and promotes autophagy. In parallel, the study confirmed the carotenoid’s potent anti-inflammatory properties, documenting a significant reduction in serum levels of the pro-inflammatory cytokines TNF-α, IL-6, and IL-1β. This effect is attributed to ASX’s ability to suppress the NF-κB inflammatory pathway, partly by activating the Nrf2 antioxidant pathway. In effect, ASX appears to break the vicious cycle in which chronic inflammation inhibits autophagy, leading to an overall improvement in the metabolic and inflammatory state of the diabetic patient.

However, when examining the stage preceding overt diabetes, namely prediabetes, the results appear more nuanced, as highlighted by the study of Ciaraldi et al. (2023) on individuals with prediabetes and dyslipidemia [143]. In this research, the primary endpoint—improvement in whole-body insulin sensitivity measured with the gold-standard euglycemic-hyperinsulinemic clamp technique—did not reach statistical significance. This result, while disappointing at first glance, does not invalidate ASX’s potential but suggests that its effects on insulin action may be more subtle or tissue-specific. Indeed, the same study noted positive trends: a tendency towards a reduction in the HOMA-IR index (a marker of insulin resistance) and an improvement in insulin sensitivity specifically at the hepatic level were observed. The liver could therefore be a primary target organ for ASX’s action, an aspect that warrants further investigation. Other studies also indirectly support a beneficial effect on glucose metabolism. In the study by Jabarpour et al. (2024) on women with PCOS, a condition characterized by strong insulin resistance, eight weeks of supplementation led to a significant reduction in fasting glucose and insulin, as well as the HOMA-IR index [145,146]. Similarly, the research by Saeidi et al. (2023) on obese men showed how the combination of physical exercise and ASX improved glucose, insulin, and HOMA-IR parameters more markedly than exercise alone [152]. Comparing these studies, it can be hypothesized that ASX’s effectiveness on glycemic control and insulin sensitivity is dose-dependent, duration-dependent, and, above all, context-dependent. In patients with diagnosed type 2 diabetes, where inflammation and autophagic dysfunction are pronounced, ASX seems to exert a robust and measurable therapeutic effect [140]. In earlier stages, such as prediabetes, the benefits may be less evident at a systemic level but still present in specific organs like the liver. Finally, in conditions of secondary insulin resistance, as in obesity or PCOS, ASX is confirmed as a valid adjuvant, especially when integrated into a multifactorial approach that includes physical activity [143].

### 4.3. Gynecological Use: Support for Fertility Through the Modulation of Cellular Stress

The field of gynecology and reproductive medicine represents one of the most innovative areas for the application of ASX, where its antioxidant and anti-inflammatory properties translate into tangible clinical benefits for complex conditions like polycystic ovary syndrome (PCOS) and endometriosis. Both pathologies are characterized by a pro-inflammatory environment and high oxidative stress, factors that compromise oocyte quality and fertility. Studies conducted by the group of Jabarpour et al. (2023, 2024) offer a comprehensive view of ASX’s mechanisms of action in women with PCOS [144,145]. In their 2024 study, they demonstrated that supplementation with 12 mg/day for eight weeks in infertile women with PCOS significantly improves systemic metabolic parameters, with a reduction in fasting glucose and insulin, the HOMA-IR index, and LDL cholesterol, along with an increase in HDL. These metabolic effects are in themselves important for ovarian function, but the same group’s 2023 research unveiled an even deeper mechanism of action at the cellular level. This study revealed that a 60-day treatment with ASX modulates endoplasmic reticulum stress (ER stress) in granulosa cells, a key pathogenetic factor in PCOS. Specifically, ASX reduced the expression of major ER stress markers, such as GRP78 and CHOP, and the mediator XBP1. Analysis of the follicular fluid confirmed its antioxidant action, showing a significant increase in total antioxidant capacity (TAC). The clinical relevance of this molecular modulation was extraordinary: while not increasing the total number of retrieved oocytes, the ASX-treated group showed a significantly higher percentage of mature (MII) oocytes, high-quality oocytes, and consequently, high-quality embryos. This suggests that ASX does not act on quantity but drastically improves the quality of the follicular microenvironment, breaking the vicious cycle between oxidative stress and ER stress and creating more favorable conditions for the development of healthy gametes.

This potential also extends to another debilitating gynecological condition, endometriosis. The study by Rostami and colleagues (2023), a randomized, triple-blind clinical trial, evaluated the effect of ASX supplementation in women with endometriosis undergoing assisted reproductive technology (ART) procedures [150]. Consistent with the results observed in PCOS, the supplementation proved effective in mitigating both oxidative stress and markers of systemic inflammation, which are pillars of endometriosis pathogenesis. Most importantly, this biochemical improvement translated into a direct and positive clinical impact on reproductive outcomes. The ASX-treated group showed a significant improvement in ART-related outcomes compared to the placebo group, suggesting that a less inflamed and oxidized environment favors higher fertilization rates, embryo quality, and pregnancy rates. Comparing the studies on PCOS and endometriosis, a common and fundamental mechanism of action emerges: ASX acts as a powerful “normalizing” agent for the pelvic and follicular microenvironment. In both conditions, characterized by excessive inflammation and free radical production, ASX neutralizes these deleterious factors. In PCOS, this translates into better metabolic function and reduced cellular stress, allowing for the maturation of higher-quality oocytes. In endometriosis, the reduction of inflammation and oxidative stress improves the overall environment for implantation and embryonic development. Both scenarios highlight how ASX is not merely a generic antioxidant but a targeted modulator that intervenes in the key pathogenetic processes of female subfertility, representing a promising and safe adjuvant strategy to improve the success rates of ART procedures in these challenging patient populations.

### 4.4. Other Studies: From Physical Exertion Response to the Management of Systemic and Local Inflammation

Beyond its applications in the metabolic and gynecological fields, ASX demonstrates remarkable versatility in a wide range of clinical and physiological contexts, primarily due to its exceptional anti-inflammatory and antioxidant properties. An area of great interest is its ability to modulate the body’s response to intense physical exercise. The study by Gonzalez et al. (2024) on professional firefighters showed that while supplementation with 12 mg/day for four weeks did not improve overall physical performance, it significantly attenuated the inflammatory response and acute physiological stress induced by exertion [141]. Specifically, lower levels of pro-inflammatory cytokines (IL-1β, IL-2, TNF-α) and the stress hormone cortisol were recorded after exercise. This suggests that ASX helps the body better manage the biochemical stress of intense activity. This connects perfectly with the study by Nieman e al. (2023), which investigated the effect of ASX on the “open window” of immunosuppression that follows strenuous exercise [151]. The results indicated that supplementation helps maintain more stable levels of certain plasma proteins crucial for immune function, which normally decrease due to oxidative stress. Read together, these two studies paint a consistent picture: ASX may not be a direct ergogenic aid (i.e., it doesn’t increase strength or endurance), but it acts as a protective agent that supports recovery by reducing inflammation and counteracting post-exercise immune suppression—a valuable benefit for athletes and workers subjected to extreme physical exertion.

This potent anti-inflammatory action has also proven effective in acute pathological contexts. The pioneering study by Youssef et al. (2025) evaluated ASX as an add-on therapy in community-acquired pneumonia (CAP) [147]. The results were remarkable: treated patients showed a substantial decrease in major systemic inflammation markers, such as interleukin-6 (IL-6), tumor necrosis factor-alpha (TNF-α), and C-reactive protein (CRP). This biochemical improvement translated into a tangible clinical benefit, with a significant improvement in SOFA and APACHE II severity scores. This study is crucial because it transfers the anti-inflammatory effect, already observed in contexts of physiological stress (Gonzalez et al., 2024) and chronic diseases to an acute infectious disease, suggesting that ASX’s ability to inhibit pathways like NF-κB can be a valuable support to standard therapies for controlling the “cytokine storm” that characterizes many severe infections [141]. ASX also demonstrates the ability to act at a local level. The study by Tian et al. (2021) on dry eye syndrome revealed that oral intake of 12 mg/day for one month significantly improved subjective symptoms (OSDI index), tear film stability (NIBUT and FBUT), and corneal epithelial integrity [149]. Interestingly, it did not increase the quantity of tears produced but improved their quality. This indicates a targeted action to reduce oxidative stress and inflammation on the ocular surface, promoting the proper functioning of the Meibomian glands and tissue repair. Finally, research continues to explore new frontiers, as highlighted by the study protocol of Mohammadi et al. (2024), which aims to evaluate the impact of ASX on patients with heart failure for the first time [142]. This study, despite its inherent limitations such as the presence of concomitant therapies, underscores the growing scientific interest in this carotenoid as a potential cardioprotective agent. In conclusion, this collection of “other studies” confirms that ASX is a pleiotropic compound whose efficacy extends from supporting athletic recovery and managing acute systemic inflammation like pneumonia, to treating localized disorders such as dry eye syndrome, paving the way for future and promising clinical applications in severe chronic diseases.

## 5. Conclusions

ASX emerges as a natural compound of considerable clinical interest due to its ability to synergistically modulate key processes related to oxidative stress, inflammation, and cellular metabolism. Available evidence shows that this carotenoid does not act merely as a simple free radical scavenger but exerts deeper regulatory effects on major molecular pathways, activating protective signaling such as Nrf2 and AMPK while inhibiting pro-inflammatory cascades like NF-κB and mTOR. These mechanisms translate into tangible systemic benefits, including improvements in glucose and lipid metabolism, insulin sensitivity, endothelial function, and overall antioxidant capacity of tissues.

ASX shows effectiveness in conditions characterized by heightened inflammation and metabolic dysfunction—such as type 2 diabetes, metabolic syndrome, obesity, PCOS, and endometriosis—where it helps restore a more balanced cellular microenvironment, enhancing oocyte quality, reproductive outcomes, and various clinical parameters. In contexts of intense physical stress, such as high-intensity athletic activity or physically demanding professions, ASX also proves valuable by reducing post-exercise inflammatory responses and supporting immune function. Moreover, its protective actions extend to acute conditions like pneumonia and to specific anatomical sites such as the ocular surface, where it helps promote tissue repair and maintain tear film stability.

Overall, ASX stands out for its favorable safety profile, biological compatibility, and ability to integrate with conventional therapeutic approaches, making it a valuable support in preserving systemic and reproductive health. Through its multi-target activity and broad applicability, it represents a promising option within integrative medicine, with significant potential to improve clinical outcomes, promote overall well-being, and strengthen physiological resilience across a wide range of conditions.

## 6. Future Limitations

Despite the encouraging findings, several limitations must be addressed to strengthen the clinical application of ASX. The considerable heterogeneity among existing studies—particularly in dosage, duration of supplementation, formulation type (esterified vs. non-esterified), and characteristics of the study populations—makes it difficult to establish standardized therapeutic protocols. Additionally, current evidence does not sufficiently clarify the compound’s real bioavailability or the individual factors that influence its absorption and metabolic utilization. Long-term data are also lacking, especially regarding safety, cumulative effects, and the impact on sustained clinical outcomes. Other fields, such as dentistry and advanced cardiovascular disease, remain underexplored, limiting the understanding of its broader therapeutic potential. Furthermore, interactions between ASX and commonly used pharmacological treatments are still poorly investigated. For these reasons, there is a clear need for larger, rigorously controlled clinical trials aimed at defining optimal dosages, mechanisms of action, and real-world benefits across more diverse and representative patient populations.

## Figures and Tables

**Table 1:** Indicators for database searches.

Article-screening strategy	**Keywords:** “astaxanthin; *Haematococcus pluvialis*; oral administration; oxidative stress; anti-inflammatory; metabolic syndrome”
	Boolean Indicators: OR and AND
	Timespan: January 2020 to July 2025
	Electronic databases: PubMed; Scopus; WOS.

**Table 2:** Summary of the studies selected and included in the systematic review, indicating authors, publication year, study design, sample characteristics, interventions, and main outcomes.

Authors	Study Type	Sample Used and Country of Study	Study Objective	Materials and Methods	Conclusions
Moqaddam et al. (2024) [139]	Randomized Controlled Trial (RCT), double-blind	60 obese males (BMI > 30 kg/m^2^), divided into 4 groups (*n* = 15 per group at the end); Iran	To explore the impact of ASX supplementation combined with CrossFit training on adipo-myokines, insulin insensitivity, and lipid levels	Duration: 12 weeks. Groups: Control, Supplement only (20 mg/day ASX), Training only (CrossFit 3x/week), Training + Supplement. Measurements of Decorin (DCN), Activin A, Myostatin, TGF-β1, Follistatin	CrossFit training with ASX reduced anthropometric, metabolic, and lipid factors. It significantly increased Follistatin and Decorin and reduced Activin A, Myostatin, and TGF-β1. The combined effect was superior to individual interventions
Sharifi-Rigi et al. (2023) [140]	Randomized, double-blind, placebo-controlled clinical trial	60 patients with Type 2 Diabetes (T2D) (*n* = 30 ASX, *n* = 30 Placebo); Iran	To determine the effects of ASX on the autophagy pathway and inflammation markers in T2D patients	Duration: 12 weeks. Dosage: 10 mg/day of ASX or placebo. Analysis of autophagy-related gene/protein expression (mTOR, Beclin-1, LC3B) in PBMCs and serum cytokines (TNF-α, IL-6, IL-1β)	ASX significantly increased the expression of Beclin-1, LC3B, Atg-5, and Atg-7 and reduced mTOR expression compared to placebo. It significantly reduced serum levels of TNF-α, IL-6, and IL-1β, suggesting an improvement in autophagy and inflammation
Gonzalez et al. (2024) [141]	Randomized double-blind, placebo-controlled, crossover study	15 career male firefighters (mean age 34.5 years); Spain	To examine the impact of ASX on oxidative stress, inflammation, cardiometabolic health, and tactical performance	Duration: 4 weeks per treatment (crossover with washout). Dosage: 12 mg/day ASX or placebo. Maximal exercise test and fire ground test (FGT). Blood/saliva analysis pre/post-exertion	ASX attenuated the ventilatory anaerobic threshold (VANT) and reduced the acute inflammatory response (IL-1β, cortisol, uric acid) to intense exercise. No significant effect on resting lipids or performance in the fire ground test
Mohammadi et al. (2024) [142]	Study Protocol (Double-blind RCT)	Planned 80 patients with heart failure (stage C and D); Iran	To evaluate the effect of ASX on inflammation, oxidative stress, lipids, blood pressure, endothelial function, and quality of life in heart failure	Planned duration: 8 weeks. Groups: Intervention (20 mg/day ASX) vs. Placebo (maltodextrin). Outcomes: Total Antioxidant Capacity (TAC), lipids, nitric oxide, quality of life questionnaires	Study protocol; results are pending. It aims to verify if AX supplementation improves total antioxidant capacity, inflammation, oxidative stress, lipid profiles, and quality of life in heart failure patients.
Ciaraldi et al. (2023) [143]	Randomized, double-blind, placebo-controlled trial	34 adults with prediabetes and dyslipidemia; United States	To determine the effects of ASX treatment on lipids, CVD markers, glucose tolerance, insulin action, and inflammation	Duration: 24 weeks. Dosage: 12 mg/day ASX or placebo. Tests: OGTT, hyperinsulinemic-euglycemic clamp, indirect calorimetry	ASX significantly reduced Total Cholesterol and LDL. It reduced cardiovascular risk markers (fibrinogen, L-selectin, fetuin-A). Trend towards improvement in insulin sensitivity, but primary endpoint not statistically reached
Jabarpour et al. (2023) [144]	Randomized controlled trial	58 infertile women with PCOS. two groups: Astaxanthin 12 mg/day for 60 days (*n* = 29) and placebo (*n* = 29). After losses to follow-up, 53 patients were analyzed (27 ASX, 26 placebo; Iran	To evaluate whether astaxanthin (ASX) supplementation can ameliorate endoplasmic reticulum (ER) stress in granulosa cells (GCs) of women with PCOS.	Procedures: standard antagonist ovarian stimulation protocol followed by ICSI. Biological analyses: Granulosa cells collected at oocyte retrieval were analyzed for gene expression (GRP78, CHOP, XBP1, ATF4, ATF6) using qPCR and protein expression using Western Blot. Follicular fluid was tested for oxidative stress markers (TAC, SOD, MDA). Clinical outcomes: Number of retrieved oocytes, MII oocyte rate, total oocyte score (TOS), fertilization rate, embryo number and quality, biochemical and clinical pregnancy rates.	reduced ER stress markers (GRP78, CHOP, XBP1); increased ATF4 and TAC, improved the MII oocyte rate, high-quality oocyte rate, and high-quality embryo rate
Jabarpour et al. (2024) [Phytother Res] [145]	Triple-blind randomized clinical trial	58 infertile women with Polycystic Ovary Syndrome (PCOS); Iran	To investigate the effect of ASX on lipid profile, insulin resistance (IR), blood pressure, and oxidative stress	Duration: 8 weeks. Dosage: 12 mg/day (2 × 6 mg) ASX or placebo. Measurement of FBS, Insulin, HOMA-IR, malondialdehyde (MDA), TAC, SOD, and lipid profile	ASX significantly reduced fasting blood sugar (FBS), HOMA-IR, Insulin, MDA and LDL cholesterol. It increased total antioxidant capacity (TAC) and HDL cholesterol. No effect on blood pressure or BMI
Jabarpour et al. (2024) [J Cell Mol Med] [146]	Randomized, double-blind clinical trial	56 women with PCOS (aged 18–40); Iran	To determine the effect of ASX on serum inflammatory markers and gene expression of endoplasmic reticulum (ER) stress and apoptosis in PBMCs	Duration: 8 weeks. Dosage: 12 mg/day ASX or placebo. Real-time PCR analysis for genes (GRP78, CHOP, etc.) and ELISA for cytokines (TNF-α, IL-6, IL-18, C-reactive protein)	ASX reduced the expression of pro-apoptotic and ER stress genes (CHOP, XBP1, ATF4, DR5) and reduced serum levels of TNF-α, IL-18, IL-6, and active caspase-3. No significant difference for C-reactive protein (CRP) or caspase-8. No clinical effect on hirsutism or BMI
Youssef et al. (2025) [147]	Prospective, randomized, double-blind, placebo-controlled study	80 adults with Community-Acquired Pneumonia (CAP); Egypt	To evaluate ASX as an adjunctive therapy on inflammatory cytokines and clinical outcomes (severity scores)	Duration: 7 days. Dosage: 12 mg/day ASX or placebo + standard antibiotic therapy. Measurements: IL-6, TNF-α, IL-10, SOFA and APACHE II scores	ASX significantly reduced pro-inflammatory cytokines (IL-6, TNF-α) compared to placebo. Severity scores (SOFA and APACHE II) improved more in the ASX group. Non-significant reduction in hospital stay
Heidari et al. (2023) [148]	Randomized, double-blind, placebo-controlled clinical trial.	50 patients with coronary artery disease (CAD) (aged 40–65); Iran	To assess the effects of astaxanthin (AX) supplementation on cardiometabolic risk factors (lipid profile, glycemic indices, anthropometric indices), SIRT1, and TNF-α levels.	Participants were randomly allocated into two groups: AX supplements (12 mg/day) or placebo for 8 weeks, along with a low-calorie diet. BMI, body composition, fasting blood sugar, insulin, lipid profile, TNF-α, and SIRT1 were measured.	AX supplementation showed no significant between-group differences for body composition, glycemic indices, or inflammation (TNF-α, SIRT1). However, significant intra-group reduction in total cholesterol and LDL-C was observed in the AX group. AX may play a beneficial role in lipid profiles, but further studies are needed.
Tian et al. (2021) [149]	Prospective, single-group, pretest-posttest quasi-experimental study.	60 middle-aged and elderly patients (120 eyes) with mild-to-moderate dry eye disease (DED); China	To evaluate the efficacy and safety of astaxanthin in the treatment of mild-to-moderate dry eye disease (DED).	Oral administration of astaxanthin tablets (12 mg total per day, divided into two doses) for 30 days. OSDI score, non-invasive tear break-up time (NIBUT/FBUT), CFS score, eyelid margin signs, and meibum quality were measured before and after treatment.	The OSDI score, tear film stability (NIBUT/FBUT), CFS score, and eyelid margin signs improved significantly after treatment. No significant changes were observed in tear quantity (Schirmer test). Oral AX was found safe and effective in improving symptoms and signs of DED.
Rostami et al. (2023) [150]	Randomized, triple-blind, placebo-controlled clinical trial.	50 infertile women with endometriosis (stage III/IV) candidates for assisted reproductive techniques (ART); Iran	To study the effect of AX on pro-inflammatory cytokines, oxidative stress markers, and early pregnancy outcomes.	Treatment with 6 mg/day of AX or placebo for 12 weeks before and during ovarian stimulation. Cytokines (IL-1β, IL-6, TNF-α) and oxidative stress markers (MDA, SOD, CAT, TAC) were measured in serum and follicular fluid, along with ART outcomes.	X significantly reduced oxidative stress (MDA) and inflammation (IL-1β, IL-6, TNF-α) and increased antioxidant capacity (TAC, SOD). Supplementation improved the number of oocytes retrieved, oocyte maturity, and embryo quality.
Nieman et al. (2023) [151]	Randomized, double-blind, placebo-controlled crossover study.	18 healthy runners (11 male, 7 female); United States	To examine the efficacy of 4-week AX ingestion in moderating exercise-induced inflammation and immune dysfunction using a multi-omics approach.	Supplementation with 8 mg/day of AX or placebo for 4 weeks prior to a 2.25-h run at 70% VO2max (including downhill running). Pre/post-exercise blood analysis for cytokines, oxylipins, and untargeted proteomics (immunoglobulins).	AX did not counter exercise-induced increases in plasma cytokines, oxylipins, or muscle soreness. However, it prevented the post-exercise decrease of 82 plasma proteins related to immune function and significantly countered the drop in immunoglobulins (IgM), providing immune support.
Saeidi et al. (2023) [152]	Randomized controlled trial (stratified into 4 groups).	68 males with obesity (BMI > 30 kg/m^2^); Iran	To investigate the effects of 12 weeks of high-intensity functional training (HIFT) with AX supplementation on adipokines (CTRP9, CTRP2, GDF8, GDF15), insulin resistance, and lipids.	Participants were divided into 4 groups: Control, Supplement only (20 mg/day AX), Training only (HIFT), and Training + Supplement. The intervention lasted 12 weeks. Metabolic profiles and specific adipokines were measured.	The combined group (Training + AX) showed the greatest improvements. Significant reductions in body weight, body fat percentage, BMI, lipid profile, and insulin resistance were observed. Adipokines CTRP9, CTRP2, GDF8, and GDF15 decreased significantly, especially in the combined group.
Supriya et al. (2023) [153]	Randomized controlled trial (stratified into 4 groups).	68 males with obesity (BMI ~33.6 kg/m^2^); Iran	To investigate the effects of 12 weeks of CrossFit training combined with AX supplementation on Semaphorin 3C (SEMA3C) and other adipokines (apelin, chemerin, omentin1, visfatin, resistin, etc.).	4 groups: Control, Supplement (20 mg/day AX), CrossFit, CrossFit + Supplement. Duration 12 weeks. Analysis of plasma levels of an extensive panel of adipokines.	The combined intervention (CrossFit + AX) produced the most pronounced results. Significant reductions were found in resistin, visfatin, apelin, RBP4, chemerin, vaspin, and SEMA3C. Significant increases in adiponectin and omentin1 were observed. AX augments the metabolic benefits of exercise.

**Table 3:** A tabular summary of the risk of bias assessment for 15 studies, evaluated across the five domains of RoB 2 (Risk of Bias 2).

Authors and Year	D1	D2	D3	D4	D5	Overall
Tian et al. (2021) [149]	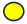	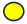	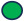	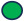	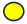	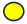
Heidari et al. (2023) [148]	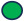	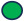	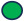	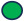	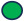	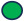
Jabarpour et al. (2023) [144]	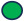	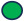	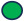	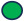	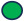	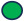
Rostami et al. (2023) [150]	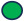	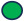	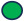	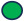	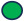	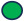
Saeidi et al. (2023) [152]	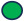	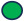	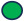	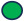	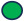	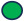
Sharifi-Rigi et al. (2023) [140]	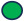	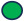	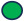	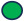	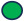	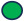
Ciaraldi et al. (2023) [143]	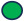	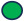	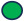	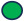	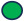	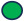
Supriya et al. (2023) [153]	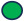	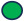	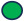	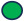	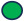	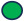
Nieman et al. (2023) [151]	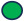	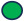	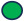	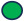	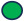	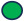
Jabarpour et al. (2023) [145]	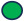	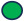	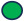	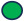	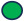	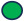
Gonzalez et al. (2024) [141]	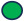	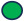	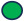	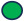	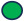	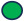
Jabarpour et al. (2024) [146]	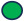	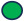	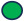	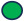	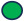	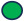
Moqaddam et al. (2024) [139]	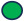	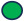	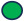	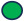	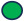	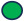
Mohammadi et al. (2024) [142]	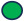	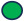	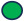	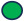	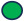	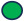
Youssef et al. (2025) [147]	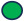	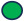	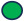	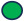	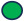	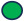

Domains: D1: Bias arising from the randomization process; D2: Bias due to deviations from intended interventions; D3: Bias due to missing outcome data; D4: Bias in the measurement of the outcome; D5: Bias in the selection of the reported result; Overall: Overall risk of bias. Judgment 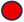: Hight; 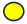: Some concerns; 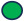: Low.

## Data Availability

The data are contained within the article.

## Abbreviations

The following abbreviations are used in this manuscript:

AMPK	AMP-Activated Protein Kinase
APACHE	Acute Physiology and Chronic Health Evaluation
ART	Assisted Reproductive Technology
ASX	Astaxanthin
BMI	Body Mass Index
CAD	Computer-Aided Diagnosis
CHOP	C/EBP Homologous Protein
CRP	C-Reactive Protein
DNA	Deoxyribonucleic Acid
ER	Endoplasmic Reticulum
HDL-C	High-Density Lipoprotein Cholesterol
IL-2	Interleukin-2
IL-6	Interleukin-6
LDL-C	Low-Density Lipoprotein Cholesterol
MDA	Malondialdehyde
NF-κB	Nuclear Factor kappa-light-chain-enhancer of activated B cells
Nrf2	Nuclear Factor Erythroid 2-Related Factor 2
PCOS	Polycystic Ovary Syndrome
ROS	Reactive Oxygen Species
SOD	Superoxide Dismutase
SOFA	Sequential Organ Failure Assessment
TAC	Total Antioxidant Capacity
TGF-β	Transforming Growth Factor-beta
TNF-α	Tumor Necrosis Factor-alpha
XBP1	X-Box Binding Protein-1

## Appendix A

### Appendix A.1. PubMed Query String

(astaxanthin OR astaxantin OR “Haematococcus pluvialis”) OR (“oral administration” OR “oral supplement*” OR “dietary supplementation”) AND (“oxidative stress” OR “reactive oxygen species” OR inflammation OR inflammatory OR “anti-inflammatory” OR metabolism OR metabolic OR “metabolic syndrome” OR glucose OR lipid);

### Appendix A.2. Scopus Query String

TITLE-ABS-KEY (astaxanthin OR astaxantin OR “Haematococcus pluvialis”) AND TITLE-ABS-KEY (“oral administration” OR “oral supplement*” OR “dietary supplementation”) AND TITLE-ABS-KEY (“oxidative stress” OR “reactive oxygen species” OR inflammation OR inflammatory OR “anti-inflammatory” OR metabolism OR metabolic OR “metabolic syndrome” OR glucose OR lipid);

### Appendix A.3. Web of Science Query String

TS = (astaxanthin OR astaxantin OR “Haematococcus pluvialis”) AND TS = (“oral administration” OR “oral supplement*” OR “dietary supplementation”) AND TS = (“oxidative stress” OR “reactive oxygen species” OR inflammation OR inflammatory OR “anti-inflammatory” OR metabolism OR metabolic OR “metabolic syndrome” OR glucose OR lipid).
